# Composition Distribution of the Thermal Soluble Organics from Naomaohu Lignite and Structural Characteristics of the Corresponding Insoluble Portions

**DOI:** 10.3390/molecules29122776

**Published:** 2024-06-11

**Authors:** Meixia Zhu, Yaya Ma, Wenlong Mo, Shihao Hao, Xianyong Wei, Xing Fan, Tiezhen Ren, Kongjun Ma, Jia Guo

**Affiliations:** 1 State Key Laboratory of Chemistry and Utilization of Carbon Based Energy Resources and Key Laboratory of Coal Clean Conversion & Chemical Engineering Process (Xinjiang Uyghur Autonomous Region), College of Chemical Engineering and Technology, Xinjiang University, Urumqi 830017, China; 18580890109@163.com (M.Z.); mowenlong@xju.edu.cn (W.M.); wei_xianyong@163.com (X.W.); fanxing@sdust.edu.cn (X.F.); rtz@xju.edu.cn (T.R.); 2 Hami Quality and Metrology Testing Institute, Hami 839000, China; hjl2595516756@163.com; 3 Key Laboratory of Coal Processing and Efficient Utilization, Ministry of Education, China University of Mining & Technology, Xuzhou 221116, China; 4 College of Chemical and Biological Engineering, Shandong University of Science and Technology, Qingdao 266590, China; 5 Xinjiang Energy Co., Ltd., Urumqi 830000, China; gj459824057@126.com

**Keywords:** naomaohu lignite, thermal dissolution, soluble organics, composition and structural characteristics

## Abstract

With cyclohexane (CH), benzene (BE), and ethyl acetate (EA) as solvents, Naomaohu lignite (NL, a typical oil-rich, low-rank coal) from Hami, Xinjiang, was thermally dissolved (TD) to obtain three types of soluble organics (NL_CH_, NL_BE_, and NL_EA_) and the corresponding insoluble portions (NL_CH-R_, NL_BE-R_, and NL_EA-R_). Ultimate analysis, X-ray photoelectron spectroscopy (XPS), Fourier transform infrared spectroscopy (FTIR), thermogravimetric analysis (TG-DTG), and gas chromatography–mass spectrometry (GC/MS) were used to characterize NL and its soluble and insoluble portions. Results showed that, compared with NL, the C element in NL_CH-R_, NL_BE-R_, and NL_EA-R_ increased, while the O element decreased significantly, indicating that thermal dissolution is a carbon enrichment process and an effective deoxidation method. The GC/MS results showed that oxygen-containing organic compounds (OCOCs) are dominant in NL_CH_, NL_BE_, and NL_EA_. NL_CH_ is mainly composed of ketones (11.90%) and esters (19.04%), while NL_BE_ and NL_EA_ are composed of alcohols (12.18% and 2.42%, respectively) and esters (66.09% and 84.08%, respectively), with alkyl and aromatic acid esters as the main components. Among them, EA exhibits significant selective destruction for oxygen-containing functional groups in NL. XPS, FTIR, and TG-DTG results showed that thermal dissolution can not only affect the macromolecular network structure of NL, but also improve its pyrolysis reactivity. In short, thermal dissolution can effectively obtain oxygen-containing organic compounds from NL.

## 1. Introduction

As petroleum resources rapidly diminish and energy demands increase, the conversion of coal into liquid fuels, chemicals, and carbon materials is garnering more attention. Among these, oil-rich coal (a kind of coal with a tar yield of 7–12% [1]) holds a significant advantage in improving oil and gas conversion efficiency and reducing economic costs [2,3]. The “Implementation Plan for Carbon Peak in the Industrial Field of Xinjiang Uygur Autonomous Region”, released on 26 July 2023, emphasizes the acceleration of the development of coal, coal-fired power, and coal chemical industry clusters; the utilization of advanced coal capacity and the enhancement of coal-to-oil and gas processes; oil-rich, low-rank coal fractionation and classification; and clean and efficient utilization. It also stresses the vigorous development of the modern coal chemical industry, the expedited construction of Zhundong and Hami national coal-to-oil and gas strategic bases, and the transformation of the coal-to-oil and gas industry towards specialty fuels, high-end chemicals, etc. Furthermore, the plan aims to develop coal-to-olefins, aromatics, and oxygen-containing compounds as basic chemical raw materials, as well as high-end polyolefins, high-performance polyesters, fibers, and other products.

The Hami region in Xinjiang is abundant in coal resources, with large reserves, diverse varieties, and easy extraction. The predicted coal resources amount to 570.8 billion tons, accounting for 12.5% of the national predicted resources and ranking first in Xinjiang. Particularly, the coal in the Naomaohu mining area of Yiwu County exhibits the “three lows and three highs” characteristics (low ash, low sulfur, low phosphorus, high calorific value, high oil content and high volatile matter). The proportion of oil-rich low-rank coal resources exceeds 90%, with oil content exceeding 10% and reaching up to 16.3% at its highest, making it a rare global resource. The oil-rich, low-rank coal in Naomaohu combines coal, oil, and gas properties, and understanding its molecular structural characteristics is crucial for its transition towards specialty fuels, high-end chemicals, and other directions.

Thermal dissolution (TD) is a technique that allows for the separation of organic matter from coal without the use of hydrogen, the addition of catalysts, operating below 400 °C and employing organic solvents [3,4,5,6]. It is also considered one of the most effective methods for efficient coal utilization. Thermal dissolution can break weak covalent bonds and medium-strength covalent bonds in coal, obtaining the soluble fraction of coal and providing insights into the composition and structure of organic matter within coal at a molecular level [7,8,9,10]. Typically, polar solvents are preferred for thermal dissolution over non-polar solvents due to their enhanced effectiveness. Masaki et al. [11] demonstrated that a strong polar solvent (N-methyl-2-pyrrolidone) can disrupt hydrogen bonds in low-rank coal, forming new hydrogen bonds with the coal, thereby increasing the thermal dissolution rate. Wang et al. [12] conducted thermal dissolution of Xianfeng lignite using various organic solvents. Among them, tetralin (a hydrogen donor solvent) plays a role in supplying hydrogen during thermal dissolution, resulting in higher dissolution yields under high-temperature conditions. Benzene, as an inert organic solvent, minimally disrupts oxygen bridge bonds in coal during thermal dissolution and does not react with the soluble fraction of coal. Therefore, the soluble fraction dissolved in benzene can reflect the basic situation of free organic compounds in coal. Zhao et al. [6] used benzene and ethanol as solvents to study the thermal dissolution of Shengli lignite (SL) and Xiaolongtan lignite (XL) at 320 °C. The results showed that, compared to XL, SL had a higher content of aromatic hydrocarbons in the soluble organic matter, with the content of aromatic hydrocarbons dissolved in benzene significantly higher than in ethanol. OCOCs were mainly ketones and phenols, with the phenol content dissolved in benzene lower than in XL, while the phenol content dissolved in ethanol was higher than in XL, indicating a lower number of free phenols in SL. Wang et al. [13] used cyclohexane as a solvent for stepwise thermal dissolution of Xianfeng lignite at 200–320 °C. The results showed that cyclohexane, due to its low boiling point, low viscosity, stable chemical properties, and non-reactivity with the coal sample during thermal dissolution, effectively extracted the organic components of Xianfeng lignite, reflecting the composition and structural characteristics of the coal sample through corresponding characterization. Li et al. [10] conducted thermal dissolution of Zhaotong lignite (ZL) in cyclohexane and methanol at 300 °C. The results indicated that cyclohexane could effectively extract the inherent components of ZL without significant cleavage of covalent bonds. Yang et al. [14] studied the thermal dissolution of SL in ethyl acetate (EA) under various conditions, such as temperature, liquid–solid ratio, and time. The results showed that, as a nucleophilic reagent, EA underwent esterification and ester exchange reactions during thermal dissolution, with EA and ethanol (produced from the reaction of EA with water in SL) significantly participating in the depolymerization of SL by attacking the C_aryl_, C_acyl_, and C_alkyl_ structures in SL. Hao et al. [15] conducted thermal dissolution of ZL and NL using ethanol, tetrahydrofuran, and ethyl acetate, and separated the thermal dissolution products into three fractions using column chromatography. The results indicated that ethanol exhibited good selectivity for phenols and esters, tetrahydrofuran released highly condensed polycyclic compounds from low-rank coal, and ethyl acetate showed strong selectivity for C=O in low-rank coal, releasing a significant amount of ester compounds during thermal dissolution.

Low-boiling organic solvents have the potential to reduce solvent loss and environmental emissions as well as enhance product yield and quality, and low-energy reaction conditions have the ability to lower production costs, energy consumption, and carbon emissions. Therefore, this study focuses on the thermal dissolution (TD) of Naomaohu lignite (NL) using cyclohexane (CH), benzene (BE), and ethyl acetate (EA) at 300 °C. The composition and structural characteristics of NL were investigated through characterization techniques, including GC/MS, ultimate analysis, FTIR, XPS, and TGA. This research aims to provide a theoretical basis for the transformation of oil-rich coal into specialty fuels and high-end chemicals.

## 2. Results and Discussion

### 2.1. Composition Distribution of NL Thermal Soluble Organics

**Figure 1:**
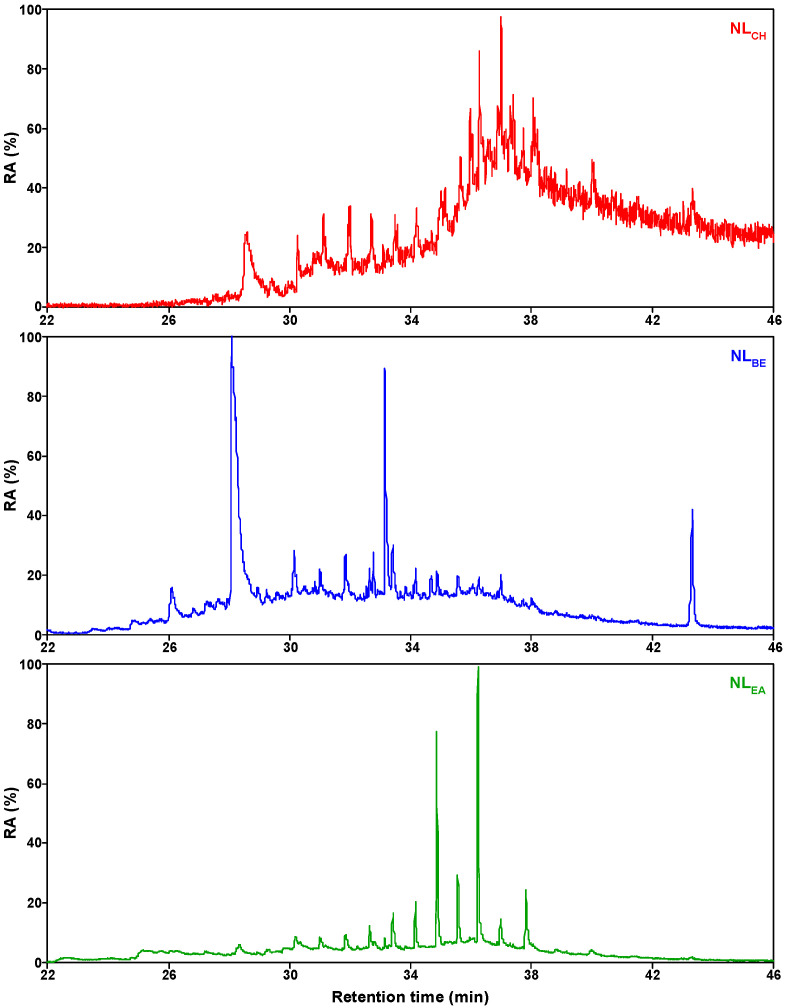
Total ion chromatograms of three thermal soluble organics (NL_CH_, NL_BE,_ and NL_EA_ are the thermal soluble substances of CH, BE, and EA, respectively).

**Figure 2:**
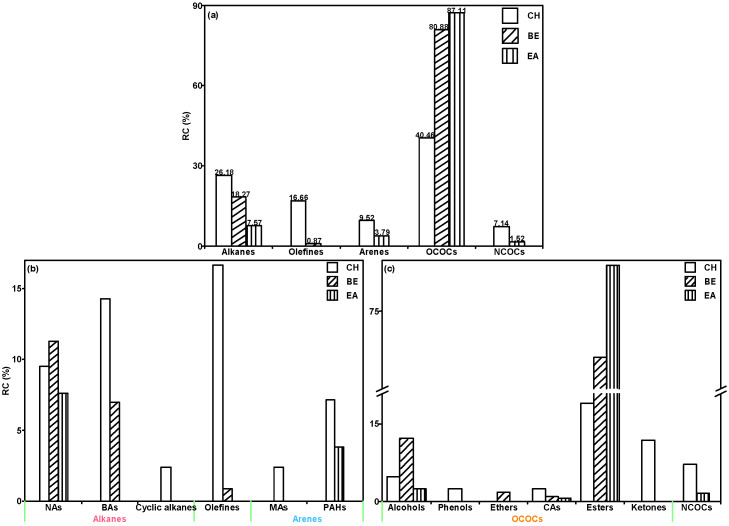
Group components distribution of three thermal soluble organics: (**a**) overall distribution diagram, (**b**) hydrocarbons, and (**c**) heteroatoms.

Figure 1 and Figures S1–S3 present the total ion chromatograms of NL_CH_, NL_BE_ and NL_EA_ (retention time 22–46 min). Figure 2 and Tables S1–S12 depict the compositional distribution of three thermally soluble organic matters. As shown in Figures S1–S3 and Tables S1–S12, GC/MS detected 33, 25, and 28 compounds in NL_CH_, NL_BE_ and NL_EA_, respectively, categorized into alkanes, olefines, aromatics, oxygen-containing organic compounds (OCOCs) and nitrogen-containing organic compounds (NCOCs). Alkanes were further classified into straight-chain, branched-chain, and cycloalkanes, while aromatics were divided into monocyclic and polycyclic aromatics. Additionally, OCOCs were categorized as alcohols, phenols, ethers, carboxylic acids, esters, and ketones, totaling 6 compound classes.

Figure 2 reveals that OCOCs dominate in NL_CH_ (40.46%), NL_BE_ (80.88%), and NL_EA_ (87.11%), indicating that small-molecule compounds with O in NL can be effectively dissociated and removed during the thermal dissolution process, releasing some small molecules (such as alkanes and aromatics). Alkane compounds follow in abundance, with the order of content being NL_CH_ (26.18%) > NL_BE_ (18.27%) > NL_EA_ (7.57%), suggesting that, as organic inert solvents, CH and BE effectively dissolve a large amount of alkane compounds in coal without reacting with the soluble portion of coal. The carbon numbers are mainly distributed in C_11_–C_25_, with the content of C_13_ alkane compounds being the highest in both NL_CH_ and NL_BE_.

As exhibited in Figure 2c, a detailed comparison of OCOCs in NL_CH_, NL_BE_, and NL_EA_ reveals that NL_CH_ is predominantly composed of ketones (11.90%) and esters (19.04%), followed by alcohols, phenols, and carboxylic acids, indicating that CH exhibits good selectivity towards ketones and esters. Specifically, ketones mainly include those containing benzene rings, pyridine ketones, and furan ketones. NL_BE_ and NL_EA_ are primarily composed of alcohols (12.18% and 2.42%) and esters (66.09% and 84.08%), respectively. It is evident that ester compounds in NL thermal dissolution products are more widely distributed and diverse in NL_EA_, possibly due to the easier cleavage of COO in NL by EA. Tables S4, S8 and S11 indicate that ester compounds in NL_CH_, NL_BE_, and NL_EA_ are mainly alkyl esters and aromatic acid esters. Notably, the content of aromatic acid esters in NL_CH_ (9.25%) is equal to that of alkyl esters, while NL_BE_ has a higher content of aromatic acid esters (62.61%), and NL_EA_ is dominated by alkyl esters (81.20%), with higher carbon numbers.

**Scheme 1:**
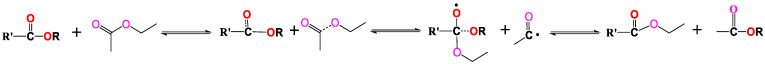
Possible pathways for the ester exchange reaction of NL during thermal dissolution (R’ represents aryl group, R represents alkyl group).

In summary, because CH and BE mainly extract the organic components present in NL during the thermal dissolution process, OCOCs in NL primarily exist in the form of free alcohols, ketones, and aromatic acid esters. However, OCOCs in NL_EA_ mainly exist in the form of alkyl esters, as EA undergoes ester exchange reactions with aromatic acid esters in NL during thermal dissolution, generating new alkyl esters, as illustrated in Scheme 1.

The aromatic acid esters in NL_BE_ mainly include 1-Butyl-2-isobutyl phthalate (BABE), di-n-butyl phthalate (DP), and Bis(2-ethylhexyl) phthalate (BEP). DP and BEP are used as plasticizers in the plastics industry to enhance flexibility and toughness of plastic products. BABE, a non-competitive α-glucosidase inhibitor with an IC50 value of 38 μM, exhibits blood glucose-lowering effects and is studied for its potential in diabetes treatment.

The thermal dissolution extraction process involves organic solvents penetrating and diffusing through the pores of coal and dissolving (exchanging) the soluble organic matter throughout the coal’s macromolecular network structure. As inert organic solvents (primarily dissolving inherent free small molecules in coal), CH and BE yield significantly different thermal dissolution products due to their distinct physical structures and solubility towards soluble organic matter in coal. CH is an alkane, while BE is an aromatic hydrocarbon, and CH’s solubility for soluble organic matter in coal (16.7 J^1/2^/cm^3/2^) is lower than that of BE (18.5 J^1/2^/cm^3/2^) [16], resulting in a higher alkane content in NL_CH_ than in NL_BE_, while the content of aromatic acid esters (the main form of free oxygen-containing organic compounds in NL) is much higher in NL_BE_ than in NL_CH_.

### 2.2. Impact of Thermal Dissolution on Element Distribution in NL

Based on Table 1, after thermal dissolution treatment, the volatile matter yield of the samples significantly increased, while the moisture and ash content decreased. Additionally, the carbon element content followed the order of NL_EA-R_ > NL_CH-R_ > NL_BE-R_ > NL, indicating that coal thermal dissolution is an effective method for carbon enrichment. Compared to NL, the H/C ratios of NL_CH-R_, NL_BE-R_ and NL_EA-R_ all decreased, with the most significant reductions observed in NL_CH-R_ and NL_BE-R_, attributed to the large amount of alkane compounds dissolved in CH and BE in the coal samples. The oxygen content in the coal samples showed the order NL_EA-R_ < NL_CH-R_ < NL_BE-R_ < NL, suggesting that thermal dissolution can effectively remove O from NL. Furthermore, compared with NL, the O/C ratio of the insoluble residues significantly decreased, providing further evidence of the deoxygenation and carbon enrichment effects of thermal dissolution treatment.

### 2.3. Changes in Functional Groups of Insoluble Portions

**Figure 3:**
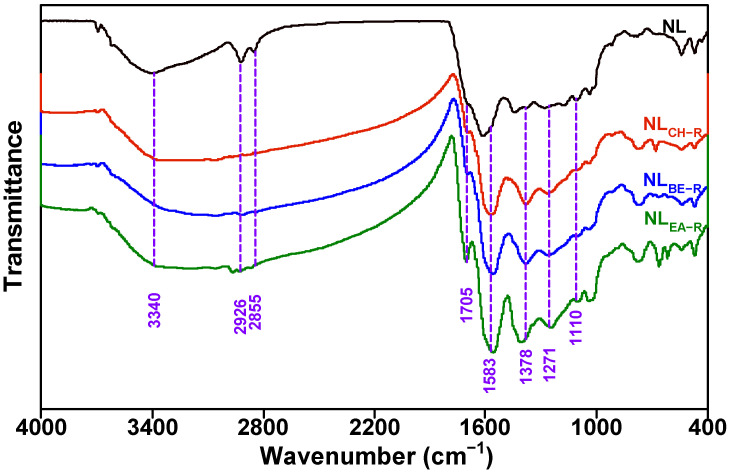
FTIR spectra of NL and its insoluble portions (NL_CH−R_, NL_BE−R_, and NL_EA−R_ are thermal insoluble substances of CH, BE, and EA, respectively).

The FTIR spectra of NL and its thermally insoluble portions, as shown in Figure 3, reveal that the peak types of NL, NL_CH-R_, NL_BE-R_, and NL_EA-R_ are generally similar, but with significant differences in peak intensities, indicating that thermal dissolution treatment affects the macro-molecular network structure of NL. The peak at 3340 cm^−1^ corresponds to the stretching vibration peak of intermolecular or intramolecular −OH bonds. Compared to NL, the peak intensities of this peak in the three thermally insoluble portions are weaker, attributed to the disruption of hydrogen bonds and other non-covalent bonds by the organic solvents used [17]. The absorption peaks near 2926 cm^−1^ and 2855 cm^−1^ are related to the stretching vibrations of −CH_2_− groups, while the peak at 1378 cm^−1^ is attributed to −CH_3_ groups. The peak intensities of these peaks in the three thermally insoluble portions are significantly lower than in NL, indicating that a large portion of the aliphatic organic compounds in NL were extracted by CH, BE, and EA during the thermal dissolution process. Moreover, the peak intensities of NL_CH-R_ and NL_BE-R_ are relatively similar, while NL_EA-R_ exhibits stronger intensities, suggesting that CH and BE have a stronger solubilizing effect on aliphatic organic compounds in NL, leading to higher levels of these compounds in NL_EA-R_ compared to NL_CH-R_ and NL_BE-R_ (consistent with the GC/MS results). The peak at 1710 cm^−1^ corresponds to the stretching vibration of conjugated C=O bonds, mainly from ketones, esters, and carboxylic acids. The strong absorption peak at 1583 cm^−1^ in all four samples indicates the presence of a significant amount of olefinic and aromatic ring structures in the coal samples. In NL_EA-R_, the peak at 1271 cm^−1^ for >CH−OH is stronger, while the intensity near 1110 cm^−1^ for >CH−O−CH< is weaker, suggesting that >CH−O−CH< bonds in NL were disrupted during the EA thermal dissolution process.

According to the Beer–Lambert Law, the intensity (area) of absorption peaks in the infrared spectrum is directly proportional to the concentration of functional groups causing the absorption if KBr pellets contain coal samples of the same concentration. In complex multi-component systems, the infrared bands often overlap, making it difficult to clearly observe the weak covalent bonds in coal. Therefore, curve-fitting analysis is used to determine individual peaks in the coal infrared spectrum [18]. This method can identify hidden peaks with fixed frequencies and half-widths but allows for changes in relative intensity. This is an important principle for obtaining accurate and reproducible results.

**Figure 4:**
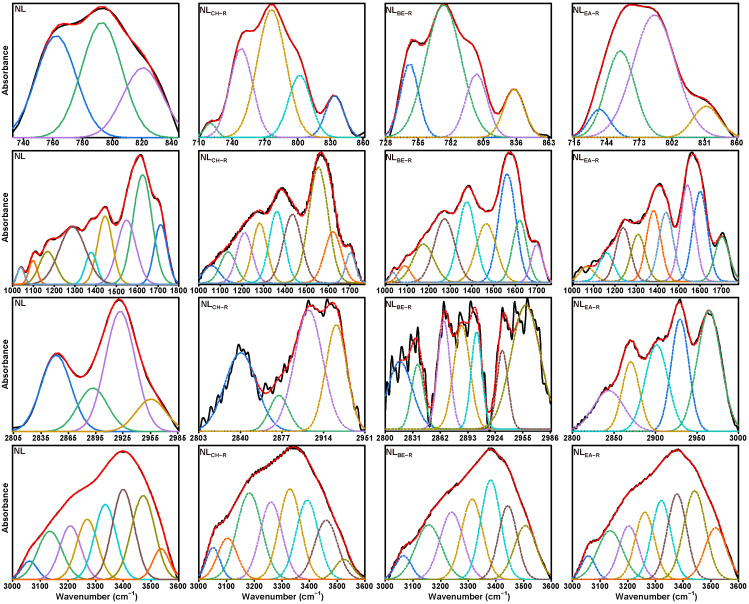
FTIR fitting results of NL and its insoluble portions (NL_CH−R_, NL_BE−R_, and NL_EA−R_ are thermal insoluble substances of CH, BE, and EA, respectively). The solid line represents the raw data, and the dashed line represents the fitted data.

As shown in Figure 4 and Table 2, the 3000–3600 cm^−1^ region represents hydroxyl absorption bands. Compared to NL (39.75%), the self-condensation −OH content (peak area percentage) in NL_CH-R_ (29.29%), NL_BE-R_ (34.79%), and NL_EA-R_ (35.18%) all decreased, indicating that CH, BE, and EA disrupted the weak hydrogen bonds in NL during thermal dissolution. The asymmetric −CH_2_ and symmetric −CH_2_ absorption peaks at 2930–2900 cm^−1^ and 2870–2810 cm^−1^ are relatively high in all samples, with contents above 20% (except for NL_BE-R_).

The ratio of the absorption peak areas of −CH_2_ to −CH_3_ can reflect the relative content of organic structures, denoted as the aliphatic structure parameter $S=A({CH}_{2})/A({CH}_{3})$ [22,23]. The values for NL (3.71) and NL_CH-R_ (1.53) are higher than those for NL_BE-R_ (0.53) and NL_EA-R_ (0.69), indicating that aliphatic hydrocarbons in NL and NL_CH-R_ mainly exist in long-chain forms with fewer side-chain aliphatic hydrocarbons. Additionally, tri-substituted benzene rings dominate in all four samples, with the relative content ranking as NL_EA-R_ > NL_BE-R_ > NL_CH-R_ > NL > 45%, indicating that hydrogen atoms on the benzene rings of the coal samples are substituted by other atoms or groups. This suggests that aromatic compounds in the coal samples may undergo a series of substitution reactions during the thermal dissolution process, leading to structural changes in the coal samples.

**Figure 5:**
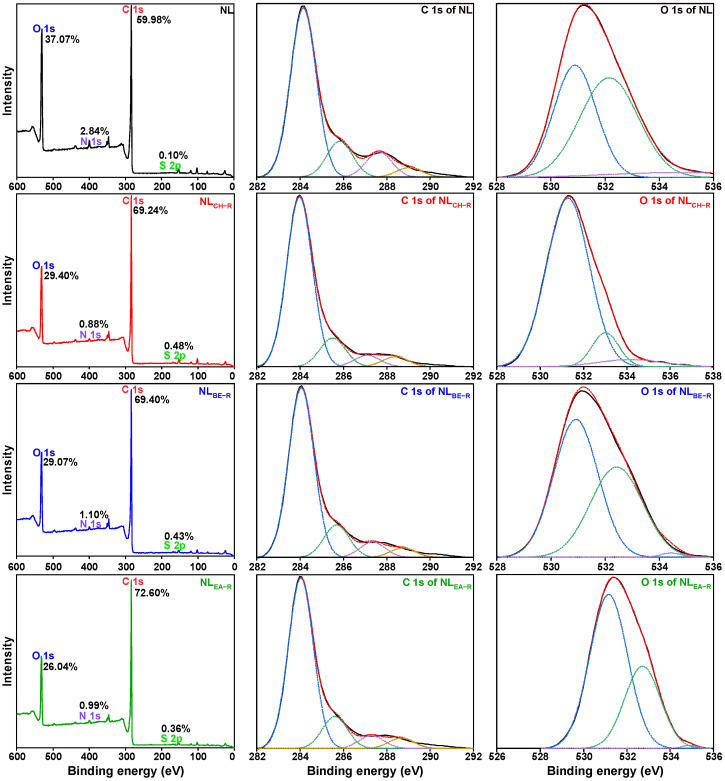
X-ray photoelectron energy spectra and fitting curves of NL and its insoluble portions (NL_CH−R_, NL_BE−R_, and NL_EA−R_ are thermal insoluble substances of CH, BE, and EA, respectively). The solid line represents the raw data, and the dashed line represents the fitted data.

Figure 5 and Table 3 present the XPS spectra of NL and its insoluble portions, as well as the relative content of various functional groups. In Figure 5, the peak intensity of C 1s increases in the order of NL < NL_CH-R_ < NL_BE-R_ < NL_EA-R_, while the peak intensity of O 1s is exactly the opposite, aligning with the elemental analysis findings. According to references [4,15,17,24,25], the binding energies of C 1s at 284.0 eV, 285.6 eV, 287.3 eV and 288.7 eV correspond to C−C, C−H, C=O, and COO, respectively. Table 3 reveals that the dominant form of C 1s on the surface of the four samples is C−C, while O 1s is mainly in the form of C=O and C−O, primarily existing as phenols, alcohols, and esters. The results of C 1s indicate that, compared to the original coal, the C=O content of NL decreased from 10.54% to 5.18%, 6.82%, and 5.67% after thermal dissolution with CH, BE, and EA, respectively, while the COO content increased from 4.01% to 4.59%, 4.20%, and 4.61%, respectively. This may be due to the destruction of oxygen bridges (such as C=O) in the coal sample during thermal dissolution in the presence of EA, leading to the formation of esters and carboxylic acid compounds [15,26]. Additionally, during the thermal dissolution processes with CH and BE at 300 °C, the capsule structure [27] in NL was disrupted, releasing some small OCOCs, with some C=O-containing compounds dissolving in CH and BE, while some COO-containing compounds were retained in the coal sample structure.

**Scheme 2:**
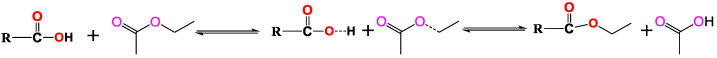
Acidolysis of EA with higher carboxylic acids (R denotes an alkyl group with more than two carbon atoms).

O 1s mainly exists in the form of C=O (531.1 eV), C−O (532.7 eV), and COO (534.5 eV) [4,15,17,24,25]. From Figure 5 and Table 3, it can be observed that, compared to NL, the C=O content increased in NL_CH-R_, NL_BE-R_, and NL_EA-R_, while the C−O and COO contents relatively decreased. The lowest C−O content was observed in NL_CH-R_ (10.60%), indicating higher content in NL_CH_ (as indicated by GC/MS, primarily existing in the form of aromatic alcohols, alkyl chain alcohols, and phenols). The COO content in NL_EA-R_ decreased from 6.08% to 0.54%, which may be due to the reaction of carboxylic acid compounds in NL with EA during the thermal dissolution process, forming ethyl acetate and acetic acid [15], with possible reaction pathways, as outlined in Scheme 2. In conclusion, the thermal dissolution treatment in the presence of CH, BE, and EA can break down and convert the oxygen-containing functional groups in NL into various forms of oxygen-containing compounds.

### 2.4. Thermal Decomposition Characteristics of Insoluble Portions

**Figure 6:**
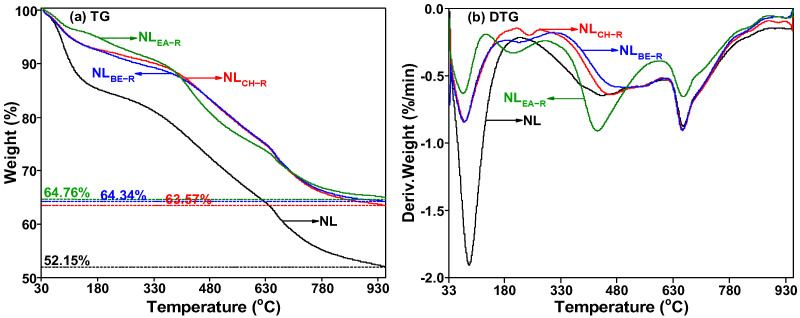
TG (**a**) and DTG (**b**) profiles of NL and its insoluble portions (NL_CH−R_, NL_BE−R_, and NL_EA−R_ are thermal insoluble substances of CH, BE, and EA, respectively).

Figure 6 shows the TG-DTG curves of NL and its insoluble portions. As depicted in Figure 6a, the four samples exhibit similar trends of mass loss, with mass losses in the order of NL (47.85%) > NL_CH-R_ (36.43%) > NL_BE-R_ (35.66%) > NL_EA-R_ (35.24%). This is attributed to the partial extraction of soluble components from NL during the thermal dissolution process by organic solvents, leading to a reduction in the mass loss of the insoluble portions.

**Figure 7:**
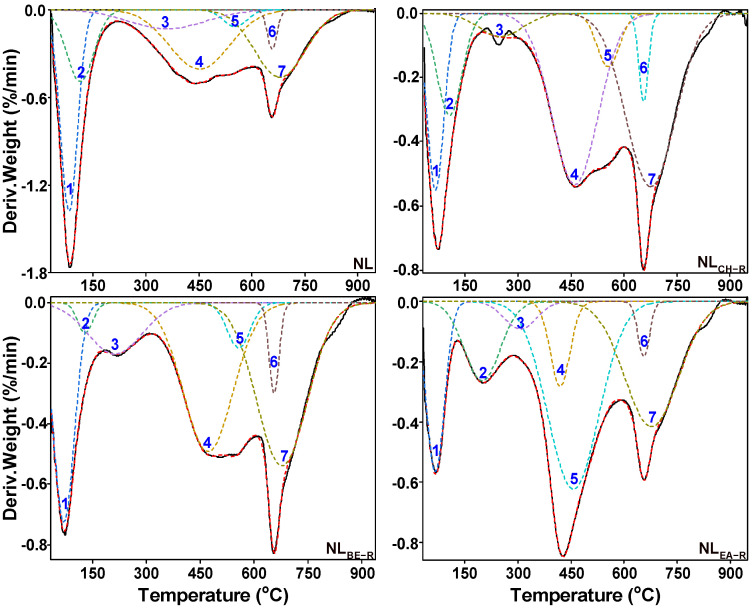
DTG fitting curve of NL and its insoluble portions (NL_CH−R_, NL_BE−R_, and NL_EA−R_ are thermal insoluble substances of CH, BE, and EA, respectively). The solid line represents the raw data, and the dashed line represents the fitted data. The corresponding explanations of the numbers are listed in Table 4.

Figure 7 and Table 4 present the DTG fitting and attribution of fitting parameters for the four samples, revealing fundamental information on the covalent bonds in NL and its insoluble portions. Combining with Figure 6b, the thermal decomposition process of the coal samples can be divided into four stages. Primarily, all four samples (especially NL) exhibit a significant peak in the mass loss rate at around 80 °C, mainly due to prolonged sample–air contact time (resulting in excessive moisture absorption). Below 400 °C (peaks 1–3), the main processes involve the volatilization of moisture and some low-boiling-point substances, as well as the release of bound water and the cleavage of carboxyl groups, accompanied by the breakage of weak bonds, such as >C_al_−N< and >C_al_−S−. Comparatively, NL_EA-R_ shows a distinct peak in the mass loss rate between 130 °C and 400 °C, attributed to the thorough swelling and loosening of the sample due to thermal dissolution treatment, promoting the escape of small molecular substances from the coal and enhancing the diffusion of some high-boiling-point organic matter within the sample.

The temperature range of 380–610 °C represents the main process of organic matter thermal decomposition in the samples, involving the cleavage of various covalent bonds leading to significant mass loss. Between 380 °C and 500 °C, the predominant bond cleavages include >C_al_−C_al_<, >C_al_−H, and >C_ar_−N, while 440–610 °C mainly involves the dissociation of strong covalent bonds, such as >C_ar_−C_al_<, >C_ar_−O-, and >C_ar_−S−. At temperatures exceeding 610 °C (peaks 6 and 7), the process primarily involves the condensation of aromatic rings and the decomposition of mineral matter, releasing some small condensed molecules, like H_2_O and CO_2_. Additionally, due to the high reaction temperature, H_2_ may also be generated during the condensation reactions of aromatic rings. In conclusion, thermal dissolution can effectively alter the thermal decomposition characteristics of coal samples (with EA exerting the most significant influence). Furthermore, selective cleavage of covalent bonds with different binding energies can be achieved by adjusting the thermal decomposition temperature, providing insights for the targeted extraction of organic matter from the samples.

## 3. Experimental

### 3.1. Raw Materials

NL was collected from the Naomaohu coal mine in Hami, Xinjiang, China. After crushing, it was sifted through a 200-mesh sieve (<74 μm) and dried naturally for 24 h. CH, BE, and EA were purchased from Tianjin Xin Platte Chemical Co., Ltd. (Tianjin, China). All the solvents are analytical reagents and were distilled in a rotary evaporator before use.

### 3.2. Thermal Dissolution

**Figure 8:**
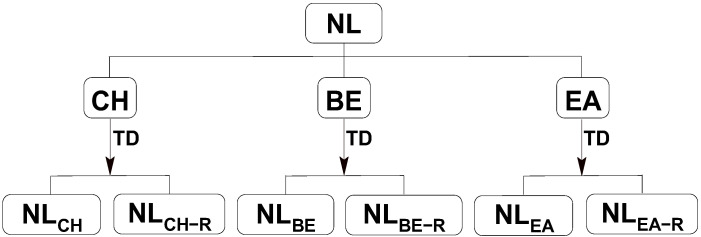
Thermal dissolution process of NL (NL_CH_, NL_BE_, and NL_EA_ are the thermal soluble substances of CH, BE, and EA, respectively; NL_CH−R_, NL_BE−R_ and NL_EA−R_ are thermal-insoluble substances of CH, BE, and EA, respectively).

NL (2 g) and CH were added to a 50 mL beaker at a solid–liquid ratio of 1:10 and thoroughly stirred before transferring to a high-pressure reaction vessel equipped with a magnetic stirrer. After purging the vessel with N_2_ to displace air, the high-pressure reaction vessel was heated to 300 °C at a rate of 20 °C/min under an initial pressure of 1 MPa and maintained for 2 h. Upon completion of the reaction, the vessel was cooled to room temperature, and the mixture was filtered to separate the filtrate and residue. The residue was washed repeatedly with CH until the filtrate became colorless and transparent, yielding CH-soluble (NL_CH_) and -insoluble (NL_CH-R_) fractions. The thermal dissolution process of NL is illustrated in Figure 8. A similar procedure was followed to obtain soluble and insoluble fractions of benzene (BE) and ethyl acetate (EA), resulting in samples designated as NL_BE_, NL_BE-R_, NL_EA,_ and NL_EA-R_, respectively.

### 3.3. Analysis Methods

The thermal dissolution products NL_CH_, NL_BE_, and NL_EA_ were analyzed using GC/MS (Thermo Fisher Scientific UltiMate 3000 UHPLC-Q Exactive, Waltham, Massachusetts, USA). The instrument was equipped with an HP-5MS chromatographic column, and high-purity helium gas (1 mL/min) was used as the carrier gas. The inlet temperature was set at 250 °C with a temperature program of 60 °C for 3 min, followed by a ramp rate of 5 °C/min up to 300 °C for 10 min. The split ratio was 10:1, and the mass scan range was set from 30 amu to 500 amu.

Ultimate analysis of C, H, N, and S content in NL and the thermally insoluble fraction was conducted using an ultimate analyzer (Elementar UNICUBE, Langenselbold, Germany), with the oxygen content calculated by the difference method. XPS (Thermo Scientific K-Alpha, Waltham, Massachusetts, USA) was employed to determine the surface elemental composition of NL and the thermally insoluble fraction. After preparing pressed pellets of the samples and placing them on the sample holder, the samples were introduced into the instrument chamber. The pressure in the chamber was maintained below 2.0 × 10^−7^ mbar, with a spot size of 400 μm, operating at 12 kV and 6 mA filament current. The full-scan pass energy was set at 150 eV, with a step size of 1 eV, while the narrow-scan pass energy was 50 eV, with a step size of 0.1 eV.

Functional groups of NL and its thermally insoluble fraction were analyzed by FTIR (Thermo Scientific Nicolet iS20, Waltham, MA, USA) at a resolution of 4 cm^−1^. The analysis was performed with 32 scans over a wavenumber range of 400–4000 cm^−1^. The thermal reactivity of the four samples was tested using a thermogravimetric analyzer (Netzsch STA 2500, Selb, Germany). The samples were heated from room temperature to 1000 °C at a rate of 10 °C/min under a nitrogen atmosphere. All fitting curves in the experiments were generated using Peakfit 4 software.

## 4. Conclusions

This study investigated the influence of three organic solvents, namely CH, BE, and EA, on the thermal dissolution characteristics of NL. Thermal dissolution treatment can extract more readily soluble organic matter from NL, thereby altering the thermal decomposition properties of the coal sample (with EA showing the most significant impact). CH and BE, acting as inert organic solvents during the thermal dissolution process, can selectively disrupt some non-covalent bonds in NL, extracting compounds such as alkanes and esters with higher carbon content. On the other hand, EA not only effectively changes the thermal decomposition characteristics of the coal sample but also dissolves a large quantity of esters in NL, primarily comprising alkyl esters and aromatic acid esters. Furthermore, at 300 °C, thermal dissolution treatment disrupts the capsule structure in NL, allowing the release of some oxygen-containing small molecular organic compounds. By effectively removing OCOCs from NL, this process may facilitate the transformation of NL into specialty fuels (coal-to-oil) and high-end chemical products (additives or intermediates for food and industrial production, and even raw materials for the production of “biodegradable plastics”).

## Figures and Tables

**Table 1:** Proximate and ultimate analyses of NL and its insoluble portions.

Sample	Proximate Analysis (wt.%)				Ultimate Analysis (daf, wt.%)					H/C	O/C
	M_ad_	A_ad_	V_daf_	FC_daf_	C	H	N	S	O^diff^		
NL	10.77	11.48	53.41	46.59	67.43	3.92	0.94	0.26	27.45	0.70	0.31
NL_CH-R_	5.83	12.75	57.86	42.14	74.91	3.21	1.03	0.26	20.59	0.51	0.21
NL_BE-R_	5.94	11.88	56.63	43.37	74.54	3.25	1.02	0.24	20.95	0.52	0.21
NL_EA-R_	5.00	10.78	67.39	32.61	75.55	3.87	0.93	0.19	19.46	0.61	0.19

_ad_: air dry basis; _daf_: dry and ash-free basis; ^diff^: by difference.

**Table 2:** Ratio of fitting peak areas of NL and its insoluble portions in various infrared spectra region [17,18,19,20,21,22].

Wavenumber/cm^−1^	Functional Group	Content (Area, %)			
		NL	NL_CH-R_	NL_BE-R_	NL_EA-R_
3600–3500	OH-π	5.15	3.39	12.50	11.12
3500–3350	Self-associated OH	39.75	29.29	34.79	35.18
3350–3260	OH-ether O	25.94	35.05	17.69	27.90
3260–3000	Cyclic OH	29.16	32.27	35.01	25.80
3000–2930	Asymmetric aliphatic −CH_3_	11.90	25.27	32.78	30.79
2930–2900	Asymmetric aliphatic −CH_2_	44.10	38.70	17.43	21.35
2900–2870	Aliphatic −CH	15.97	7.89	15.42	21.38
2870–2810	Symmetric aliphatic −CH_2_	28.03	28.14	34.37	26.49
1700	Carboxylic acids C=O	8.00	3.68	5.34	8.63
1650	Conjugated C=O	14.37	10.61	10.78	17.50
1600–1480	C=C in ARs	29.34	27.32	22.63	18.74
1480–1400	Asymmetric −CH_3_, −CH_2_	13.03	15.41	14.65	13.39
1400–1240	Symmetric −CH_3_	4.76	24.22	33.48	22.85
1240–1160	Phenols C−OH	19.50	9.47	9.83	10.43
1160–1090	Grease C−O	7.96	5.91	2.48	5.60
1090–1030	Alkyl ethers	3.05	3.38	0.80	2.86
860–810	Four adjacent H deformation	24.31	9.56	11.87	9.06
810–750	Three adjacent H deformation	40.12	62.89	72.10	84.39
750–720	Two adjacent H deformation	35.57	27.55	16.03	6.55

**Table 3:** Distribution of C and O forms in NL and its insoluble portions.

Elemental Peak	Functionality	Binding Energy (eV)	Molar Content (%)			
			NL	NL_CH-R_	NL_BE-R_	NL_EA-R_
C 1s	C−C	284.0	70.45	77.19	74.68	75.55
	C−H	285.6	14.99	13.04	14.30	14.17
	C=O	287.3	10.54	5.18	6.82	5.67
	COO	288.7	4.01	4.59	4.20	4.61
O 1s	C=O	531.1	42.41	84.06	56.16	65.94
	C−O	532.7	51.51	10.60	43.03	33.52
	COO	534.5	6.08	5.35	0.81	0.54

**Table 4:** Relative content of functional groups for NL and its insoluble portions [28,29,30].

Peak	Possible Origin	PTR (°C)
1–3	Cleavage of weak bonds (e.g., >C_al_−O−, >C_al_−N<, and >C_al_−S−), release of combined water and decarboxylation	<400
4	Breakage of relatively strong bonds (e.g., >C_al_−C_al_<, >C_al_−H, >C_al_−O and >C_ar_−N)	380–500
5	Dissociation of strong bonds (e.g., >C_ar_−C_al_<, > C_ar_−O− and >C_ar_−S−)	440–610
6–7	CO_2_ release from carbonate decomposition and condensation of aromatic rings to release H_2_	>610

PTR: Peak temperature range.

## Data Availability

The original contributions presented in the study are included in the article/Supplementary Materials, further inquiries can be directed to the corresponding authors.

## Supplementary Materials

The following supporting information can be downloaded at: https://www.mdpi.com/article/10.3390/molecules29122776/s1, Figure S1: Total ion chromatogram of NL_CH_; Figure S2: Total ion chromatogram of NL_BE_; Figure S3: Total ion chromatogram of NL_EA_; Tables S1–S5: Composition distribution of thermal soluble organics in NL_CH_; Tables S6–S8: Composition distribution of thermal soluble organics in NL_BE_; Tables S9–S12: Composition distribution of thermal soluble organics in NL_EA_; Table S13: Group components distribution of NL_CH_, NL_BE_, and NL_EA_ with GC/MS analysis.
