# Supplementary material for: Composition Distribution of the Thermal Soluble Organics from Naomaohu Lignite and Structural Characteristics of the Corresponding Insoluble Portions
Source: Molecules. 2024 Jun 11;29(12):2776. doi: 10.3390/molecules29122776 (PMC11207049; doi:10.3390/molecules29122776)
Supplement: Supplementary file 1 [file molecules-29-02776-s001.zip › molecules-2947427-supplementary.pdf]

Composition distribution of the thermal soluble organics from Naomaohu lignite and structure characteristics of the corresponding insoluble portions

ZHU Meixia [1], MA Yaya [1,*], MO Wenlong [1], HAO Shihao [2], WEI Xianyong [1,3], FAN Xing [1,4], REN Tiezhen [1], MA Kongjun [1,*], GUO Jia [5]

[1] *State Key Laboratory of Chemistry and Utilization of Carbon Based Energy Resources and Key Laboratory of Coal Clean Conversion & Chemical Engineering Process (Xinjiang Uyghur Autonomous Region), College of Chemical Engineering and Technology, Xinjiang University, Urumqi 830017, Xinjiang, China;*

[2] *Hami Quality and Metrology Testing Institute, Hami 839000, Xinjiang, China;*

[3] *Key Laboratory of Coal Processing and Efficient Utilization, Ministry of Education, China University of Mining & technology, Xuzhou 221116, Jiangsu, China;*

[4] *College of Chemical and Biological Engineering, Shandong University of Science and Technology, Qingdao 266590, Shandong, China;*

[5] *Xinjiang Energy Co., LTD, Urumqi 830000, Xinjiang, China.*

Corresponding Author: Ma Yaya, E-mail: mayy@qibebt.ac.cn; Ma Kongjun, E-mail: Kjma@xju.edu.cn.

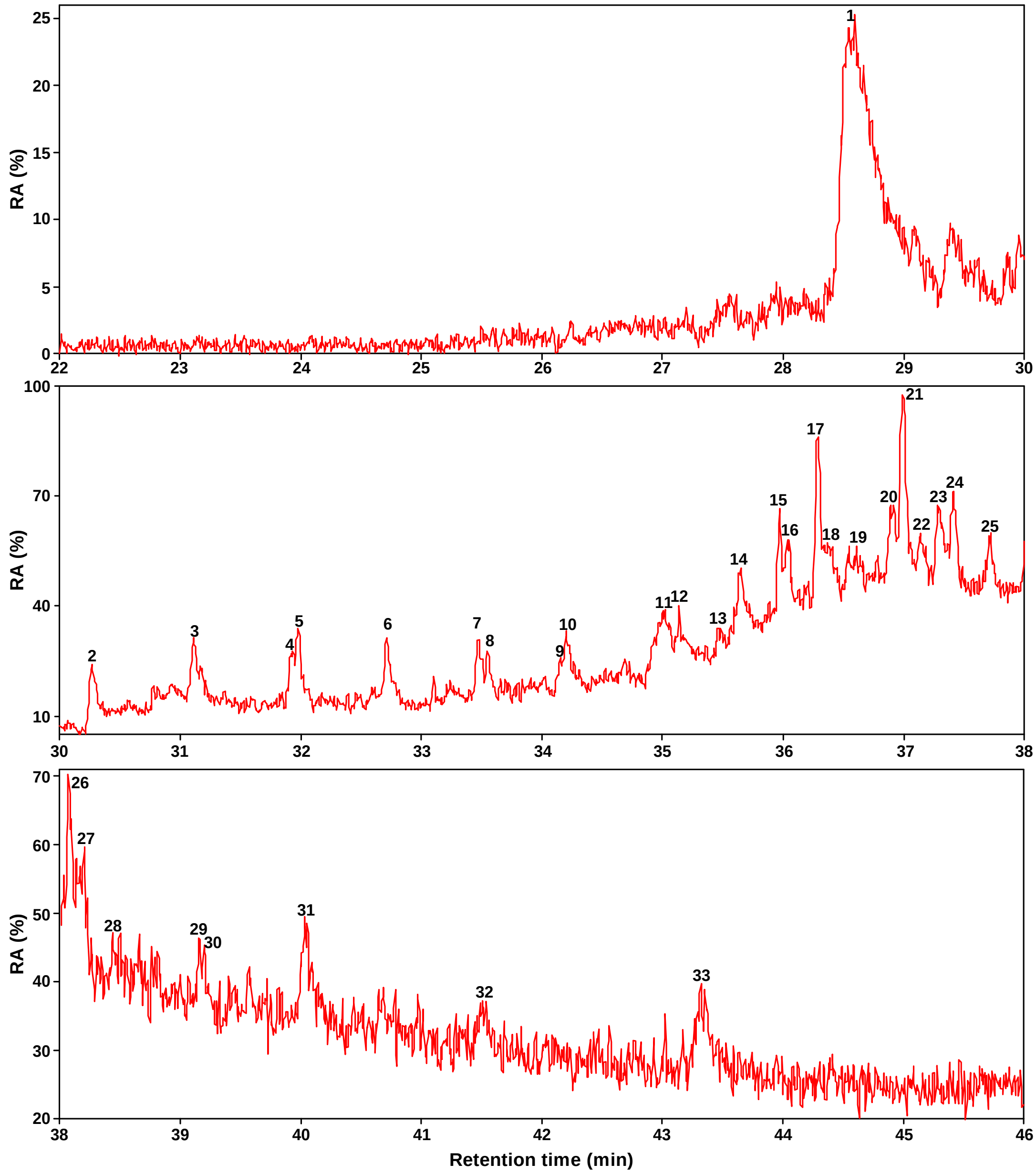

Fig. S1 Total ion chromatogram of NL_{CH}

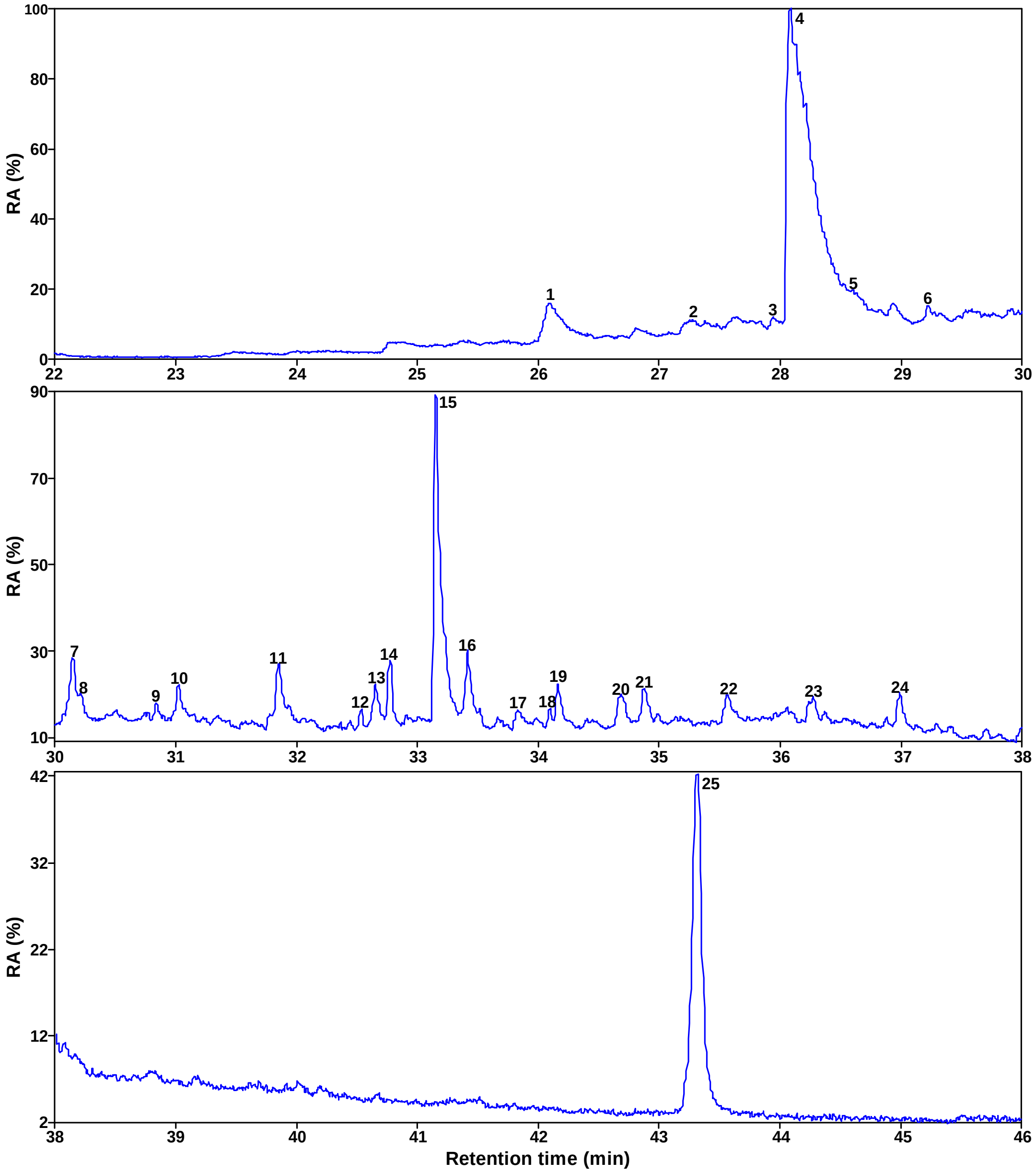

Fig. S2 Total ion chromatogram of NL_{BE}

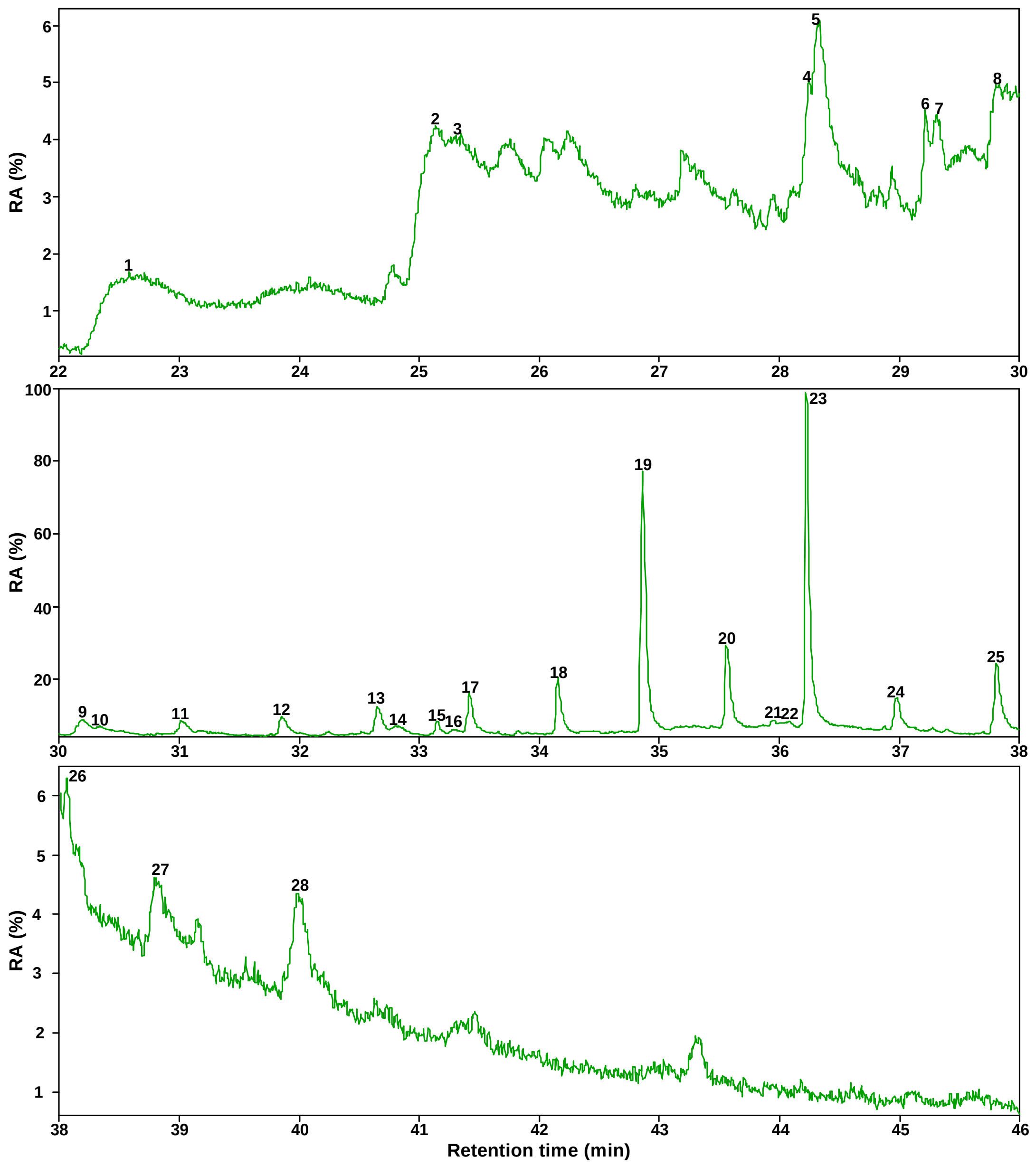

Fig. S3 Total ion chromatogram of NL_{EA}

Table S1

Alkanes detected in NL_{CH}

Species	Peak	Compound	CAS	RC (area %)
Normal alkanes (NAs)	**3**	Tridecane	000629-50-5	2.38
	**8**	Hexadecane	000544-76-3	2.38
	**10**	Heptadecane	000629-78-7	4.76
Branched alkanes (BAs)	**2**	Decane, 3-methyl-	013151-34-3	2.38
	**4**	Decane, 2,3,5-trimethyl-	062238-11-3	2.38
	**5**	Nonane, 2-methyl-5-propyl-	031081-17-1	2.38
	**6**	Undecane, 2,9-dimethyl-	017301-26-7	2.38
	**11**	Hexadecane, 2-methyl-	001560-92-5	4.76
Cyclic alkanes	**24**	1,2-Di-but-2-enyl-cyclohexane	1000188-05-3	2.38

Table S2

Olefines detected in NL_{CH}

Peak	Compound	CAS	RC (area %)
**9**	(E)-2-Hydroxy-4'-cyano-stilbene	1000147-85-5	2.38
**13**	Cyclopentene, 1-isopropyl-2,3-dimethyl-	007712-73-4	2.38
**15**	1-Methyl-4-(1-methylethyl)-3-[1-methyl-1-(4-methylpentyl)-5-methylheptyl]cyclohexene	1000370-41-4	2.38
**19**	Decane, 5,6-bis(2,2-dimethylpropylidene)-, (E,Z)-	055712-56-6	2.38
**23**	18,19-Bisnor-5-cholene, 3,24-dihydroxy-	1000251-69-1	4.76
**29**	trans-3,4,5-Trimethoxy-.beta.-methyl-.beta.-nitrostyrene	038059-94-8	2.38

Table S3

Arenes detected in NL_{CH}

Species	Peak	Compound	CAS	RC (area %)
Monocyclic aromatics (MAs)	**20**	Naphthalene, 6-(1-ethylpropyl)-1,2,3,4-tetrahydro-	054889-56-4	2.38
Polycyclic aromatic hydrocarbon (PAHs)	**26**	Anthracene, 9-ethyl-	000605-83-4	4.76
	**30**	Benzene, 1,1'-(2-cyclopropen-1-ylidene)bis-	022825-21-4	2.38

Table S4

Oxygen-containing organic compounds detected in NL_{CH}

Species	Peak	Compound	CAS	RC (area %)
Alcohols	**14**	2,2-Dimethyl-6-methylene-1-[3,5-dihydroxy-1-pentenyl]cyclohexan-1-perhydrol	1000212-02-6	4.76
Phenols	**25**	Phenol, 2,6-bis(1,1-dimethylethyl)-	000128-39-2	2.38
Carboxylic acids (CAs)	**22**	2-Propenoic acid, 3-(1H-indol-3-yl)-	001204-06-4	2.38
Esters	**1**	Phthalic acid, decyl 2-methoxyethyl ester	1000315-80-6	4.76
	**7**	Oxalic acid, 2-ethylhexyl isohexyl ester	1000309-38-8	2.38
	**16**	Methyl 2-hydroxydodecanoate	051067-85-7	2.38
	**17**	Isophthalic acid, allyl propyl ester	1000345-64-2	4.76
	**18**	Hexadecanoic acid, 4-nitrophenyl ester	001492-30-4	2.38
	**33**	1,2,4-Benzenetricarboxylic acid, 4-dodecyl dimethyl ester	033975-29-0	2.38
Ketones	**21**	Pyridazin-3(2H)-one, 4-(1-benzotriazolyl)-6-(2-furoyl)-2-phenyl-	256421-54-2	7.14
	**28**	3(2H)-Benzofuranone, 6-methoxy-2-[(4-methoxyphenyl)methylene]-, (E)-	036685-48-0	2.38
	**31**	Cyclohexane-1,3-dione, 2-allylaminomethylene-5,5-dimethyl-	104926-37-6	2.38

Table S5

Nitrogen-containing organic compounds detected in NL_{CH}

Peak	Compound	CAS	RC (area %)
**12**	2,6-Dimethyl-3,4-bis(trimethylsilyloxymethyl)pyridine	1000079-52-1	2.38
**27**	N-Methyl-1-adamantaneacetamide	031897-93-5	2.38
**32**	2-Amino-4-hydroxy-6,8-dimethyl-7(8H)-pteridinone	025477-64-9	2.38

Table S6

Alkanes detected in NL_{BE}

Species	Peak	Compound	CAS	RC (area %)
Normal alkanes (NAs)	**1**	Tridecane	000629-50-5	4.93
	**2**	Hexadecane	000544-76-3	0.29
	**6**	Pentadecane	000629-62-9	0.29
	**11**	Tetracosane	000646-31-1	2.90
	**13**	Eicosane	000112-95-8	1.45
	**19**	Nonadecane	000629-92-5	1.45
Branched alkanes (BAs)	**3**	Dodecane, 2,6,10-trimethyl-	003891-98-3	0.29
	**8**	Pentadecane, 2-methyl-	001560-93-6	0.87
	**16**	Heptadecane, 2,6,10,15-tetramethyl-	054833-48-6	2.61
	**21**	2-methylhexacosane	1000376-72-7	1.16
	**22**	Heptadecane, 2,6,10,15-tetramethyl-	054833-48-6	1.16
	**23**	2,6,10,14-Tetramethyl-7-(3-methylpent-4-enylidene) pentadecane	1000370-41-6	0.87

Table S7

Olefines detected in NL_{BE}

Peak	Compound	CAS	RC (area %)
**18**	3-Eicosene, (E)-	074685-33-9	0.29
**9**	1-Docosene	001599-67-3	0.58

Table S8

Oxygen-containing organic compounds detected in NL_{BE}

Species	Peak	Compound	CAS	RC (area %)
Alcohols	**10**	Ethanol, 2-(octadecyloxy)-	002136-72-3	0.87
	**12**	1-Dodecanol, 2-octyl-	005333-42-6	0.29
	**20**	Ethanol, 2-(hexadecyloxy)-	002136-71-2	1.45
	**25**	1,4-Epoxynaphthalene-1(2H)-methanol, 4,5,7-tris(1,1-dimethylethyl)-3,4-dihydro-	056771-86-9	9.57
Ethers	**14**	9-Octadecene, 1,1-dimethoxy-, (Z)-	015677-71-1	1.74
Carboxylic acids (CAs)	**17**	2-methyloctacosane	1000376-72-8	0.87
Esters	**4**	1,2-benzenedicarboxylic acid-1-butyl ester-2-iso-butyl	017851-53-5	48.70
	**5**	Dibutyl phthalate	000084-74-2	1.74
	**7**	Oxalic acid, cyclobutyl pentadecyl ester	1000309-70-5	2.32
	**15**	Bis(2-ethylhexyl) phthalate	000117-81-7	12.17
	**24**	E-8-Methyl-9-tetradecen-1-ol acetate	1000130-81-4	1.16

Table S9

Alkanes detected in NL_{EA}

Species	Peak	Compound	CAS	RC (area %)
Normal alkanes (NAs)	**6**	Heptadecane	000629-78-7	0.45
	**11**	Hexadecane	000544-76-3	1.52
	**12**	Pentadecane	000629-62-9	2.27
	**13**	Heneicosane	000629-94-7	3.33

Table S10

Arenes detected in NL_{EA}

Species	Peak	Compound	CAS	RC (area %)
Polycyclic aromatic hydrocarbon (PAHs)	**8**	Fluoranthene	000206-44-0	0.76
	**10**	Benzene, 1,1'-(1,3-butadiyne-1,4-diyl)bis-	000886-66-8	1.97
	**21**	Benzo[a]pyrene	000050-32-8	1.06

Table S11

Oxygen-containing organic compounds detected in NL_{EA}

Species	Peak	Compound	CAS	RC (area %)
Alcohols	**14**	1,2,10-Trihydroxyanthracene	000577-33-3	1.97
	**26**	(1S,2E,4S,5R,7E,11E)-Cembra-2,7,11-trien-4,5-diol	1000140-92-3	0.45
Carboxylic acids (CAs)	**1**	Benzoic acid, 4-ethoxy-	000619-86-3	0.61
Esters	**2**	1,3-Benzenedicarboxylic acid, diethyl ester	000636-53-3	0.15
	**3**	1,4-Benzenedicarboxylic acid, diethyl ester	000636-09-9	0.91
	**4**	Diethyl Phthalate	000084-66-2	0.61
	**5**	Undecanoic acid, 2,8-dimethyl-, methyl ester	055955-74-3	2.12
	**7**	Ethyl tridecanoate	028267-29-0	0.61
	**9**	Octadecanoic acid, ethyl ester	000111-61-5	3.48
	**15**	Phthalic acid, di(2-propylpentyl) ester	1000377-93-5	1.21
	**16**	Ethyl 13-docosenoate(ethyl erucate)	037910-77-3	0.76
	**17**	Docosanoic acid, ethyl ester	005908-87-2	4.39
	**18**	Eicosanoic acid, ethyl ester	005908-87-2	4.55
	**19**	Ethyl tetracosanoate	024634-95-5	18.33
	**20**	Nonanoic acid, 2,6-dimethyl-, methyl ester	055955-67-4	7.12
	**23**	Tetracosanoic acid, 2,4,6-trimethyl-, methyl ester	055335-01-8	25.45
	**24**	11-Dodecenoic acid, 2,4,6-trimethyl-, methyl ester, (R,R,R)-(-)-	030459-92-8	4.55
	**25**	Heptadecanoic acid, ethyl ester	014010-23-2	8.33
	**27**	5-Acetoxy-3-methyl-hexanoic acid, methyl ester	1000192-54-4	0.45
	**28**	Ethyl 9-hexadecenoate	054546-22-4	1.06

Table S12

Nitrogen-containing organic compounds detected in NL_{EA}

Peak	Compound	CAS	RC (area %)
**22**	Benzenamine, 4-octyl-N-(4-octylphenyl)-	000101-67-7	1.52

Table S13

Group components distribution of NL_{CH}, NL_{BE} and NL_{EA} with GC/MS analysis

**Group component**	
**Full name**	**Nomenclature**
Relative abundance (%)	RA
Relative content (area %)	RC
Oxygen-containing organic compounds	OCOCs
Normal alkanes	NAs
Branched alkanes	BAs
Polycyclic aromatic hydrocarbon	PAHs
Carboxylic acids	CAs
Nitrogen-containing organic compounds	NCOCs
Naomaohu lignite	NL
Cyclohexane	CH
Benzene	BE
Ethyl acetate	EA
Thermally soluble portions of CH, BE and EA	NL_{CH}, NL_{BE}, NL_{EA}
Thermally insoluble residues of CH, BE and EA	NL_{CH-R}, NL_{BE-R}, NL_{EA-R}
Fourier transform infrared spectroscopy	FTIR
Thermogravimetric analysis	TG-DTG
x-ray photoelectron spectroscopy analysis	XPS
Gas chromatography-mass spectrometry	GC/MS
